# Combining electrocardiographic criteria for predicting acute total left main coronary artery occlusion

**DOI:** 10.3389/fcvm.2022.936687

**Published:** 2022-08-11

**Authors:** Chunwei Liu, Fan Yang, Yuecheng Hu, Jingxia Zhang, Ximing Li, Zhigang Guo, Yin Liu, Hongliang Cong

**Affiliations:** ^1^Department of Cardiology, Tianjin Chest Hospital, Tianjin, China; ^2^Tianjin Medical University, Tianjin, China; ^3^Department of Diagnostic Ultrasound, National Clinical Research Center of Cancer, Key Laboratory of Cancer Prevention and Therapy, Tianjin Medical University Cancer Institute and Hospital, Tianjin, China; ^4^Department of Cardiac Surgery, Tianjin Chest Hospital, Tianjin, China

**Keywords:** left main, ECG, collateral circulation, ST elevation, aVR

## Abstract

**Background:**

Prediction of left main artery (LM) occlusion may contribute to the administration of early reperfusion. We sought to identify electrocardiographic (ECG) features associated with acute total LM occlusion and explore the relationship between ECG features and collateral circulation.

**Methods:**

We retrospectively studied ECGs in 84 consecutive patients with LM occlusion between January 2001 and April 2022. The ECG findings in these patients were compared with those in 468 consecutive patients with LM subtotal occlusion and non-LM occlusion.

**Results:**

Three main ECG patterns were described according to the characteristics of ST elevation (STE) in LM occlusion: ST-segment elevation myocardial infarction (STEMI), STE in aVR with diffuse ST depression, and STE in both aVR and aVL. These ECG patterns were associated with different collateral filling territories. One-third STEMI in LM occlusion showed STE in the precordial leads including V1, while 2/3 STEMI showed STE in the precordial leads from V2 to V5 without STE in V1. The following ECG characteristics predicted LM occlusion: STE in both aVR and aVL; STE in I, aVL, and V2–V5 without V1; left anterior fascicular block (LAFB); right bundle branch block (RBBB) + LAFB; and prolongation of the QRS interval. The incidences of STE in aVR and STE in aVR and V1 were higher in LM subtotal occlusion than in LM occlusion. The combination of two different STE criteria (STE in aVR and aVL and STE in I, aVL, V2-V5 without V1) predicted LM occlusion with 62% sensitivity and 95% specificity. The combination of the STE criteria and fascicular block criteria (LAFB and LAFB + RBBB) further improved the specificity to 99% but reduced the sensitivity to 39%.

**Conclusion:**

The combination of STE criteria predicted LM occlusion with high specificity and moderate sensitivity, and the addition of fascicular block criteria further improved the specificity with some loss of sensitivity.

## Graphical Abstract

Combining ECG criteria for prediction of LM occlusion, *p < 0.05. 
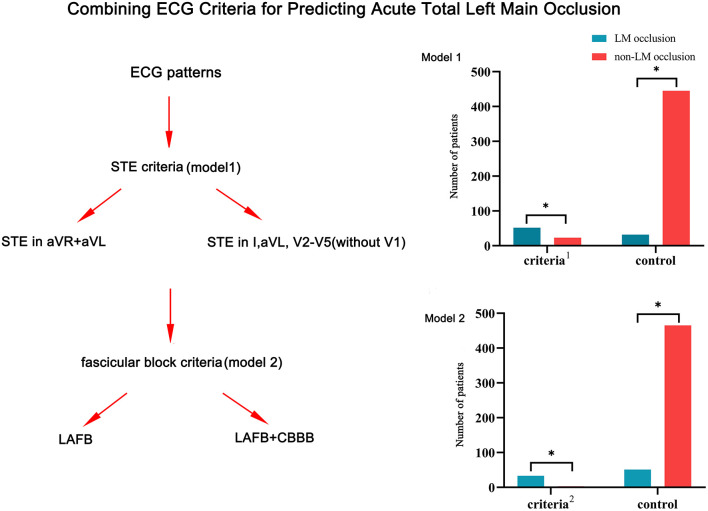

## Introduction

Acute total left main coronary artery (LM) occlusion is a rare, yet lethal disorder that often results in abrupt and severe cardiogenic shock, lethal arrhythmias, and sudden cardiac death. The 12-lead electrocardiogram (ECG) is an important tool in facilitating the identification of this LM culprit disease (1). Different ECG patterns associated with acute total LM occlusion, including ST elevation (STE) myocardial infarction (STEMI) and non-STEMI (NSTEMI), have been reported in small series (2–4). The STEMI pattern (STE in leads I, aVL, and V2–V6) corresponds to total LM occlusion without collateral circulation (5), and the NSTEMI pattern (widespread ST depression in ≥7 leads with STE in the aVR and V1) corresponds to subtotal occlusion (6). Previous studies have been largely confined to small cohorts and/or mixed patients with total and subtotal occlusions; therefore, comprehensive knowledge of different ECG characteristics that are suggestive of LM is currently limited. Furthermore, a standardized description of ECG criteria for LM occlusion has not yet been developed (7). Our previous study demonstrated the association between ECG patterns and collateral filling territories in a small cohort of patients with total LM occlusion (8). In the current study, we retrospectively compared ECG findings in patients with LM occlusion to those in patients with LM subtotal occlusion and non-LM occlusion and defined specific ECG criteria that distinguished these entities. Additionally, we aimed to investigate the association between ECG patterns and collateral circulation in patients with LM occlusion.

## Methods

### Study population

We retrospectively analyzed 17,670 consecutive patients who presented with acute myocardial infarction (AMI) and were admitted to Tianjin Chest Hospital, Tianjin, China, between January 2001 and April 2022. Eighty-four patients with total LM occlusion (visible thrombus and 100% stenosis) were included in the study. The 12-lead ECG findings in these patients were compared with those in 468 consecutive patients with non-LM occlusion between January 2019 and April 2022: 89 with LM subtotal occlusion (TIMI1-3 and >90% stenosis), 85 with left anterior descending coronary artery occlusion proximal to the first septal and first diagonal (pLAD), 98 with middle LAD occlusion (mLAD), 91 with left circumflex artery (LCX) occlusion, and 105 with right coronary artery (RCA) occlusion. Patients with a history of Q-wave myocardial infarction or coronary artery bypass graft surgery were excluded. The study was conducted in accordance with the Declaration of Helsinki, and the protocol was approved by the Ethics Committee of the Institute of Tianjin Chest Hospital. Signed informed consent was obtained from all individuals. All the data supporting the findings of this study are available from the corresponding author upon reasonable request.

### Electrocardiography and angiography

The ECG closest to the angiography was used for analysis by two independent cardiologists (CWL and HLC) when more than one ECG was available. ST elevation was defined as follows: STE ≥ 1 mm in all leads other than leads V2–V3 and STE ≥ 2 mm for men and ≥1.5 mm for women in leads V2–V3 (9). Coronary angiography was reviewed by two independent operators (JXZ and YCH), and collateral circulation was classified into four types (LAD, LCX, both LAD and LCX, and no collateral circulation) according to the territory supplied by collateral flow.

### Statistical analyses

SPSS Statistics for Windows, Version 25, software (IBM) was used to perform all data management and analysis. Data are presented as mean ± SD for continuous variables and as the proportion of valid cases for discrete variables. Differences in prevalence between groups were compared using chi-square analyses, and the mean values of continuous variables were compared using one-way ANOVA. A two-tailed *p*-value of <0.05 was considered statistically significant.

## Result

### Incidences of STE and intraventricular conduction delay in LM occlusion

**Figure 1:**
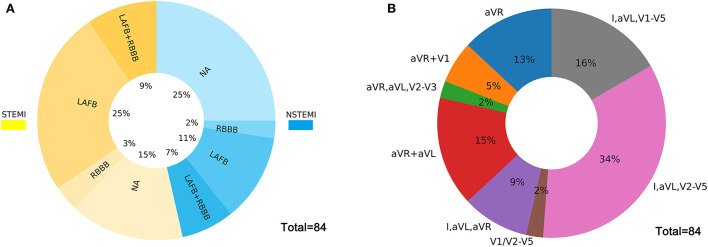
**(A)** illustrates the incidences of intraventricular conduction delay in STEMI and NSTEMI. **(B)** illustrates the incidences of different STE patterns in the LM occlusion group. LM, left main coronary artery; STE, ST-segment elevation; STEMI, ST-segment elevation myocardial infarction; NSTEMI, non-ST-segment elevation myocardial infarction; RBBB, right bundle branch block; LAFB, left anterior fascicular block.

**Figure 2:**
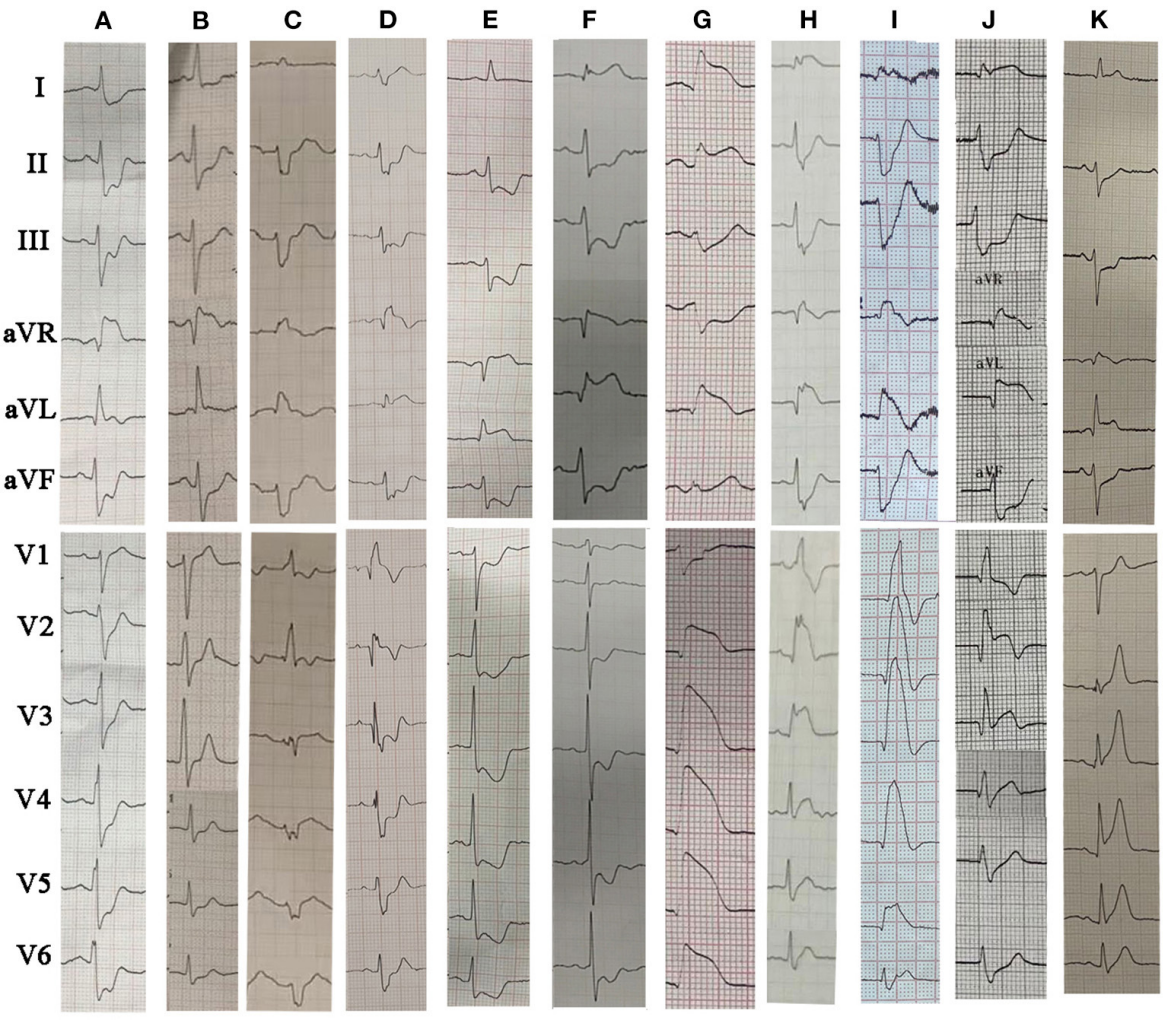
The representative 12-lead ECGs for different ECG patterns in LM occlusion. **(A)** STE in aVR; **(B)** STE in aVR+V1; **(C)** STE in aVR plus LAFB + RBBB; **(D)** STE in aVR + aVL plus RBBB; **(E)** STE in aVR + aVL; **(F)** STE in I, aVL and aVR plus LAFB; **(G)** STE in I, aVL, V2–V6; **(H)** STE in I, aVL, V2–V6 plus LAFB + RBBB; **(I)** STE in I, aVL, V1–V6 plus LAFB + RBBB; **(J)** STE in I, aVL, aVR and V2–V3 (collateral filling of the LAD territory); **(K)** STE in I, aVL, aVR, V2–V6 plus LAFB. LM, left main coronary artery; STE, ST-segment elevation; RBBB, right bundle branch block; LAFB, left anterior fascicular block.

The ECGs were divided into three main patterns according to the characteristics of STE in LM occlusion: (i) STEMI pattern: STE in the precordial leads from V1/V2 to V4 through V6 and in the lateral leads I and avL; (ii) aVR pattern: STE in aVR or in both aVR and V1 without STE in precordial leads, concomitant with widespread ST-segment depressions in the inferior leads and V4–V6; and (iii) aVR + aVL pattern: STE in both aVR and aVL or in I, aVL, and aVR without STE in V4–V6. Figure 1 illustrates the incidence of right bundle branch block (RBBB), left anterior fascicular block (LAFB), bifascicular blocks, and different STE patterns in LM occlusion. LAFB, RBBB, and LAFB + RBBB occurred in 37, 6, and 17% of total LM occlusions, respectively. LAFB was present in 47% of patients with STEMI and 26% of patients with NSTEMI (*p* < 0.05). There was only one case of the left bundle branch block in the LM occlusion. The most frequent STE pattern for LM occlusion was STE in I, aVL, and V2–V5 (29, 34%), followed by STE in I, aVL, and V1–V5 (14, 16%), STE in aVR and aVL (13, 15%), STE in aVR (11, 13%), STE in I, aVL, and aVR (8, 9%), and STE in aVR and V1 (5, 5%). Figure 2 shows representative 12-lead ECGs for different ECG patterns.

### Comparison between the LM occlusion and LM subtotal occlusion groups

Within a median follow-up time of 75 months (interquartile range: 23–143 months), the overall incidence of mortality was 51.8%. The probability of survival at 43 months was 51.7 ± 5.6%. Patients with LM occlusion were younger and had an increase in risks of in-hospital mortality by four-folds, compared to patients with LM subtotal occlusion (Table 1). Patients with LM occlusion presented with more STE in aVR and aVL, STEMI, LAFB and LAFB + RBBB, prolongation of QRS interval, less STE in aVR, and less STE in aVR and V1 (Table 1, *p* < 0.01). Of these ECG findings, STE in both aVR and aVL predicted LM occlusion with a specificity of 96% and a sensitivity of 27%. LAFB + RBBB predicted LM occlusion with a specificity of 100% and a sensitivity of 17%.

**Table 1:** Comparison of baseline characteristics and ECG findings in different groups.

	**LM occlusion (*n =* 84)**	**LM subocclusion (*n =* 89)**	**pLAD occlusion (*n =* 85)**	**mLAD occlusion (*n =* 98)**	**LCX occlusion (*n =* 91)**	**RCA occlusion (*n =* 105)**	***p*-value**
Age	61 ± 12	65 ± 10	63 ± 13	63 ± 11	62 ± 14	64 ± 11	0.246
Male	73 (87%)	61 (69%)	63 (74%)	74 (76%)	69 (76%)	84 (80%)	0.097
Hypertensio*n*	39 (46%)	45 (51%)	41 (48%)	51 (52%)	36 (40%)	44 (42%)	0.473
Diabetes	24 (29%)	29 (33%)	22 (26%)	28 (29%)	23 (25%)	31 (30%)	0.909
Onset-to-FMC (h)	3.9 ± 2.8	4.6 ± 2.7	4.1 ± 2.4	4.6 ± 2.6	4.8 ± 2.5	4.6 ± 2.7	0.161
In-hospital mortality	37 (44%)	10 (11%)	8 (9%)	4 (4%)	1 (1%)	1 (1%)	<0.001
NSTEMI	39 (46%)	83 (93%)	12 (14%)	6 (6%)	14 (15%)	7 (7%)	<0.001
STE in aVR	11 (13%)	53 (60%)	5 (6%)	4 (4%)	8 (9%)	7 (7%)	<0.001
STE in aVR+V1	5 (6%)	26 (29%)	4 (5%)	2 (2%)	3 (3%)	0 (0%)	<0.001
STE in aVR+aVL	23 (27%)	4 (5%)	3 (4%)	0 (0%)	3 (3%)	0 (0%)	<0.001
STEMI	45 (54%)	6 (7%)	73 (86%)	92 (94%)	77 (85%)	98 (93%)	<0.001
STE in V1–V5	1 (1%)	1 (1%)	35 (41%)	42 (43%)	0 (0%)	0 (0%)	<0.001
STE in V2–V5	1 (1%)	0 (0%)	0 (0%)	2 (2%)	0 (0%)	0 (0%)	0.241
STE in I, aVL, V1–V5	14 (17%)	3 (3%)	32 (38%)	21 (21%)	0 (0%)	0 (0%)	<0.001
STE in I, aVL, V2–V5	29 (35%)	2 (2%)	6 (7%)	5 (5%)	0 (0%)	0 (0%)	<0.001
STE in II, III, aVF	0 (0%)	0 (0%)	0 (0%)	2 (2%)	65 (71%)	98 (93%)	<0.001
QRS interval (ms)	117 ± 23	93 ± 13	96 ± 18	98 ± 18	99 ± 17	99 ± 15	<0.001
HDAVB	0 (0%)	0 (0%)	0 (0%)	1 (1%)	1 (1%)	13 (12%)	<0.001
RBBB	5 (6%)	1 (1%)	7 (8%)	10 (10%)	1 (1%)	3 (3%)	0.012
LBBB	1 (1%)	0 (0%)	1 (1%)	1 (1%)	1 (1%)	0 (0%)	0.822
LAFB	31 (37%)	11 (12%)	7 (8%)	13 (13%)	3 (3%)	0 (0%)	<0.001
STE in aVR, aVL + LAFB	5 (6%)	1 (1%)	1 (1%)	0 (0%)	0 (0%)	0 (0%)	0.002
STE in I, aVL, V2–V5 + LAFB	16 (19%)	0 (0%)	1 (1%)	0 (0%)	0 (0%)	0 (0%)	<0.001
LAFB+RBBB	14 (17%)	0 (0%)	1 (1%)	0 (0%)	0 (0%)	0 (0%)	<0.001
STE in aVR, aVL + LAFB + RBBB	6 (7%)	0 (0%)	0 (0%)	0 (0%)	0 (0%)	0 (0%)	<0.001
STE in I, aVL, V2–V5 + LAFB + RBBB	6 (7%)	0 (0%)	0 (0%)	0 (0%)	0 (0%)	0 (0%)	<0.001

LM, left main coronary artery; pLAD, proximal left anterior descending coronary artery; mLAD, middle left anterior descending coronary artery; LCX, left circumflex artery; RCA, right coronary artery; STE, ST-segment elevation; STEMI, ST-segment elevation myocardial infarction; NSTEMI, non-ST-segment elevation myocardial infarction; Onset-to-FMC, symptoms onset to first medical contact; HDAVB, high-degree atrioventricular block; RBBB, right bundle branch block; LBBB, left bundle branch block; LAFB, left anterior fascicular block.

### Comparison between the LM occlusion and LAD occlusion groups

There was no significant difference in the baseline characteristics between the LM occlusion and LAD occlusion groups. Proximal LAD occlusion was more related to lower in-hospital mortality than LM occlusion, similar to the mortality of LM subtotal occlusion. STE in the precordial leads, including V1, was observed in 92% of STEMI associated with pLAD occlusion. In contrast, only 1/3 STEMI in LM occlusion showed STE in the precordial leads, including V1, and 2/3 STEMI showed STE in the precordial leads from V2 to V5 without STE in V1 (Table 1, *p* < 0.01). Anterior and lateral STEMI (STE in I, aVL, and precordial leads) were present in 96% of STEMI cases due to LM occlusion, compared to 27% of the STEMI cases due to mLAD occlusion and 45% of the STEMI cases due to pLAD occlusion. Moreover, STE in I, aVL, and V2–V5 along with LAFB distinguished LM occlusion without collateral circulation from LAD occlusion with a specificity of 99% and a sensitivity of 36%.

### Comparison between the LM occlusion, LCX occlusion, and RCA occlusion groups

STE in the inferior leads (II, III, and aVF) was present in 71% of LCX occlusions and 93% of RCA occlusions. In contrast, ST depression in the inferior leads was observed in 92% of the LM occlusions. In patients with LCX occlusion, the incidences of lateral STEMI (STE in V5–V6, I, and aVL), inferior STEMI (STE in II, III, and aVF), posterior STEMI (STE in V7–V9), inferior and posterior STEMI (STE in II, III, aVF, and V7–V9) were 7, 24, 7, and 47%, respectively. Compared with LM occlusion, patients with RCA occlusion presented with greater incidences of bradycardia and high-degree atrioventricular block.

### Combining ECG criteria for prediction of LM occlusion

**Figure 3:**
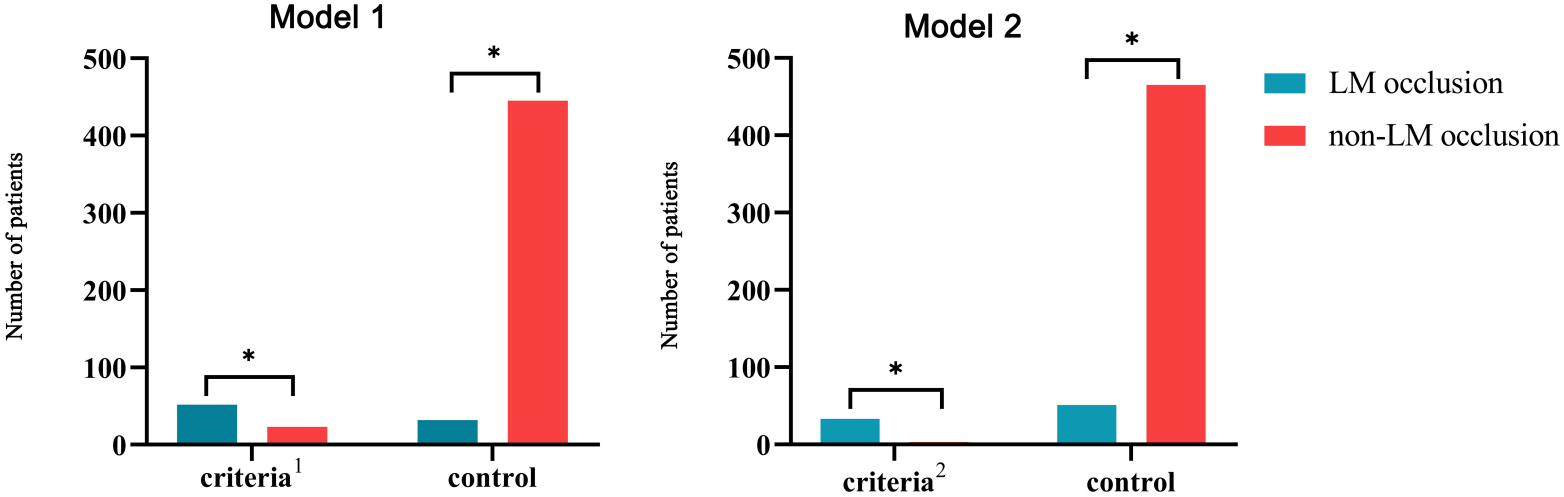
Combining ECG criteria for the prediction of LM occlusion. Model 1: Combination of two STE criteria (STE in both aVR and aVL and STE in I, aVL, V2–V5 without V1); Model 2: combination of two STE criteria and two fascicular block criteria (STE in both aVR and aVL plus LAFB, STE in both aVR and aVL plus LAFB + RBBB, STE in I, aVL, V2–V5 plus LAFB, and STE in I, aVL, V2–V5 plus LAFB + RBBB). LM, left main coronary artery; STE, ST-segment elevation; RBBB, right bundle branch block; LAFB, left anterior fascicular block. *p < 0.05.

Due to the limited sensitivity of a single ECG criterion, two types of combined ECG criteria were developed for the detection of LM occlusion (Figure 3). Model 1 included two STE criteria: STE in both aVR and aVL and STE in I, aVL, V2–V5 without V1. The combined criteria distinguished LM occlusion from non-LM occlusion with 62% sensitivity and 95% specificity. Model 2 included four ECG criteria: STE in both aVR and aVL plus LAFB; STE in both aVR and aVL plus LAFB + RBBB; STE in I, aVL, V2–V5 plus LAFB; and STE in I, aVL, V2–V5 plus LAFB + RBBB. The combination of STE and fascicular block criteria improved the diagnostic specificity up to 99% but reduced the diagnostic sensitivity to 39%.

The ROC curve was used to evaluate the predictive ability of LM occlusion. The cutoff value for the QRS interval was 107 ms (AUC, 0.757; sensitivity, 69%; specificity, 78%).

### Relation between ECG features and collateral circulation in LM occlusion

**Figure 4:**
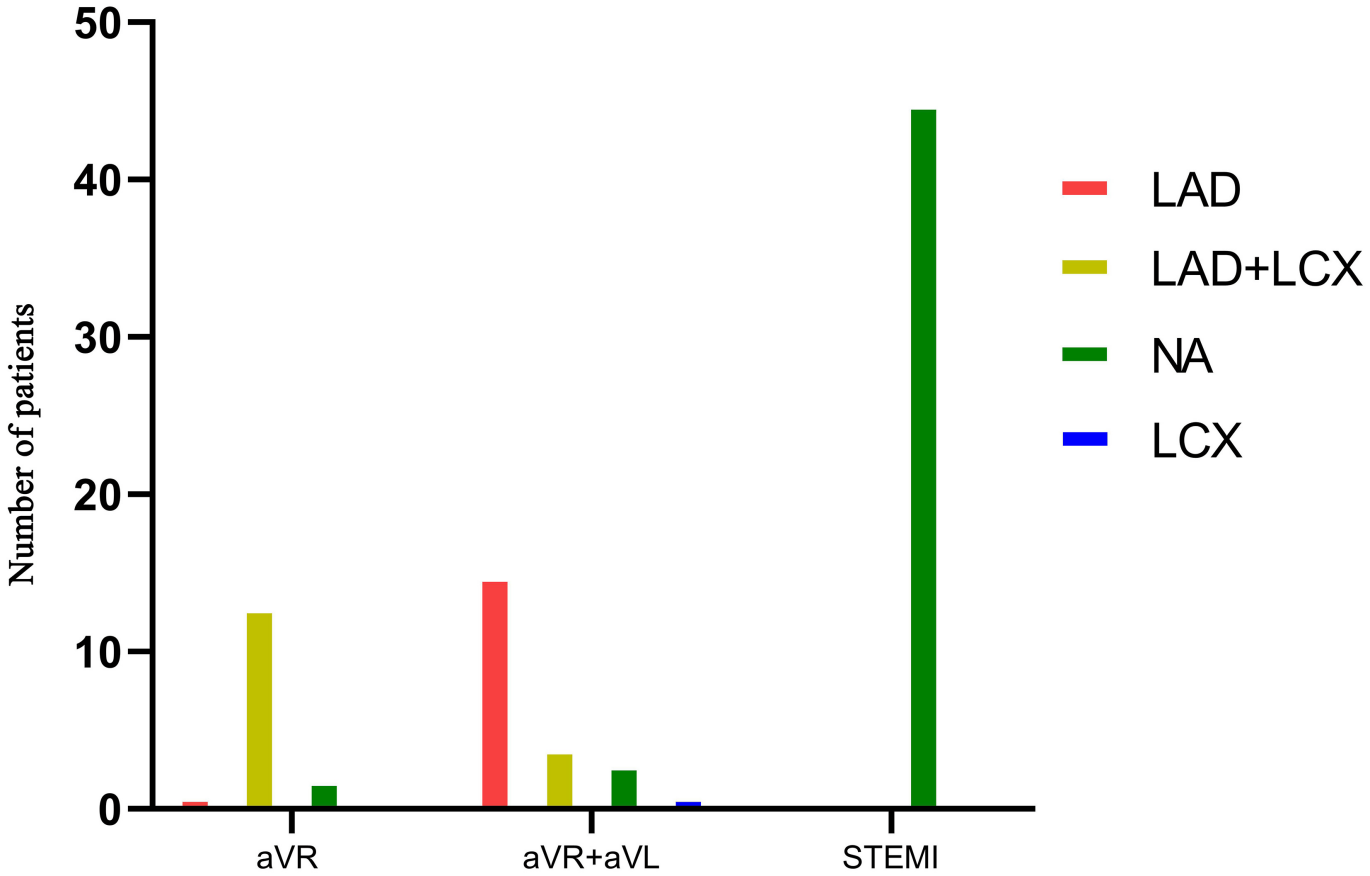
Relationship between collateral filling territories and the three main ECG patterns in LM occlusion group.

**Figure 5:**
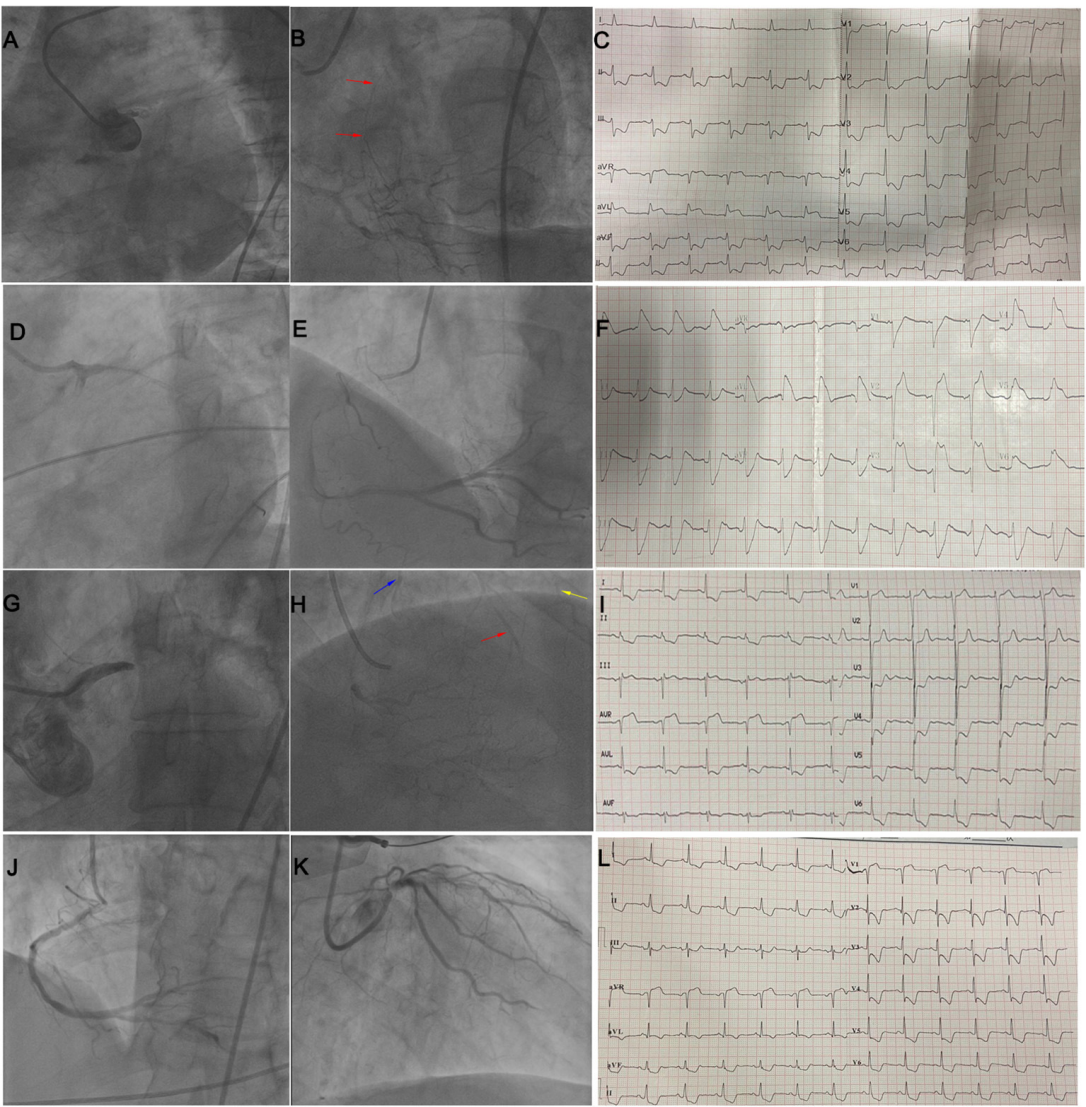
**(A–C)** illustrate STE in both leads aVR and aVL in a patient with LM occlusion and collateral filling of LAD (red arrow). **(D–F)** illustrate STE in I, aVL, V2–V5 in a patient with subacute stent thrombosis in LM and no collateral circulation. **(G–I)** illustrate STE in lead aVR in a patient with LM occlusion and collateral filling of the LAD (red arrow), diagonal branch (yellow arrow), and LCX (blue arrow). **(J–L)** illustrate STE in leads aVR and V1 in a patient with subtotal LM occlusion and no collateral circulation. LM, left main; STE, ST-segment elevation; LAD, left anterior descending coronary artery; LCX, left circumflex artery.

Thirty-four patients presented with collateral circulation from the contralateral RCA in LM occlusion (Rentrop score ≥1). Figure 4 illustrates the relationship between the collateral filling territories and the three main ECG patterns. STEMI predicted the absence of collateral flow with a specificity of 100%, whereas NSTEMI predicted collateral flow with a specificity of 90% (Table 2). Moreover, collateral filling of the LAD territory was observed in 65% of patients with STE in both aVR and aVL, whereas collateral filling of both LAD and LCX territories was observed in 81% of patients with STE in lead aVR (Figure 5, *p* < 0.01). Patients with collateral circulation presented with less LAFB than patients without collateral circulation (*p* < 0.05). However, there was no significant difference in the QRS interval between the collateral circulation groups.

**Table 2:** Relation between ECG patterns and collateral circulation in LM occlusion.

**ECG feature**	**Collateral circulation (*n =* 34)**	**Non-collateral circulation (*n =* 50)**	***P***
STE in aVR	10 (29%)	1 (2%)	<0.001
STE in aVR+V1	4 (12%)	1 (2%)	0.063
STE in aVR+aVL	20 (59%)	3 (6%)	<0.001
STEMI	0 (0%)	45 (90%)	<0.001
QRS	112 ± 25	120 ± 22	0.097
LAFB	7 (21%)	24 (48%)	0.011
RBBB	2 (6%)	3 (6%)	0.982
LAFB+RBBB	5 (15%)	9 (18%)	0.691

LM, left main coronary artery; STE, ST-segment elevation; STEMI, ST-segment elevation myocardial infarction; RBBB, right bundle branch block; LAFB, left anterior fascicular block.

## Discussion

To the best of our knowledge, this is the largest study to describe ECG patterns in patients with acute total LM occlusion (TIMI grade 0). Three main ECG patterns (STEMI pattern, STE in aVR with widespread ST depression, and STE in both aVR and aVL) were found in LM occlusion. The variety of ECG presentations caused by different collateral filling territories results in limited sensitivity in predicting LM occlusions using a single ECG criterion. Therefore, we first developed two new types of combined ECG criteria. The combination of two different STE criteria (STE in both aVR and aVL and STE in I, aVL, V2–V5 without V1) increased the sensitivity of the ECG, compared with using either criterion alone. The combination of STE criteria and fascicular block criteria (LAFB and LAFB + RBBB) further improved the diagnostic specificity, albeit with some loss in sensitivity (GRAPHICAL ABSTRACT).

STE in aVR with diffuse ST depression has been demonstrated to be a powerful ECG predictor of significant LM stenosis/multivessel disease (10, 11) and associated with a higher 30-day mortality in STEMI patients (12). Previous studies have demonstrated that STE in aVR may be absent in 20–38% of LM occlusions and present in almost 25% of LAD occlusions proximal to the first septal branch (13, 14). Our study demonstrated that isolated STE in aVR with diffuse ST depression was neither sensitive nor specific for total LM occlusion but was more common in LM subtotal occlusion. Because STE in lead aVR with widespread ST depression indicated global subendocardial ischemia during acute coronary syndrome (15), it is reasonable to hypothesize that this STE deviation could be observed in two types of LM with similar extents of subendocardial ischemia: subtotal occlusion and total occlusion with well-developed collateral circulation (collateral filling of both the LAD and LCX territories). Similarly, STE in aVR and V1 is a specific criterion for LM subtotal occlusion rather than for LM occlusion. This finding corroborated with a previous report that showed that STE in aVR and V1 is characteristic of LM subtotal occlusion (16). The incidence of STE in aVR and V1 in our study was significantly lower than that reported by Kurisu et al. (17) for LM occlusion (6 vs. 40%), which may have been observed due to the small number of patients with the NSTEMI pattern in the study by Kurisu et al.

STE in both aVR and aVL has been reported to be a specific predictor in discriminating LM occlusion from LM subtotal occlusion (16) and pLAD occlusion (17), which was consistent with our study. Takayuki et al. reported that STE at aVL rather than aVR predicted in-hospital mortality in patients with left main acute coronary syndrome (18). As shown in a previous study (19), STE in aVL is associated with lateral ischemia in the occlusion of the first diagonal or the first obtuse margin. It is reasonable to assume that STE in both aVR and aVL reflects the vector of injury current counteracting STE in the precordial leads, toward the superior direction in the frontal plane, during collateral filling of the LAD territory without the LCX territory.

Previous studies have demonstrated that STE in V1 is associated with LM and pLAD lesions during exercise tolerance testing (20), and small conal branches of the RCA did not reach the interventricular septum in acute LAD occlusion (21). In the case of LAD occlusion proximal to the first septum, the injury vector pointed rightward due to basal septum involvement, causing STE in V1 (22). On the other hand, in the case of LM occlusion without collateral circulation, occlusion of the LCX resulted in STE in the lateral leads and ST depression in the right precordial leads, counteracting the STE in V1 caused by occlusion of the LAD. A previous study demonstrated that simultaneous LAD + LCX occlusion damped the STE induced by single LAD occlusion in leads V1 in a swine model (23). Several cases of LM occlusion have described this STEMI pattern resembling pLAD occlusion, with STE in the precordial leads V2–V5 but not in V1 (24–27). The current study is the first to demonstrate that STE in the precordial leads from V2–V5, without V1, is a specific predictor for distinguishing LM and LAD occlusion. However, it is important to note that 1/3 STEMI in LM occlusion presents with STE in the precordial leads including V1, and the electrophysiological mechanism of whether ST at lead V1 is elevated in LM occlusion remains elusive.

A proximal LAD occlusion may result in a new LAFB, because the left anterior fascicle receives its blood supply from the first major septal branch of the LAD (28). LAFB was demonstrated in 80% of the patients with acute total LM occlusion in a previous study with a small patient series (17) and in 54% of the patients (including LAFB + RBBB) in our study. The high incidence of LAFB in LM occlusion indicated significant ischemia of the anterior wall, especially in the absence of collateral circulation. Since RBBB and LAFB receive essentially the same blood supply from the proximal LAD, LM occlusion may result in the coexistence of LAFB and RBBB (29). Bifascicular block (LAFB + RBBB) was reported to be one of the two main ECG features in LM occlusion (14). The present study found isolated RBBB and LAFB + RBBB in 6 and 17% of the patients with LM occlusion, respectively, which is in good agreement with the incidence reported by Petr Widimsky et al. in 35 patients with acute LM occlusion (30).

This study had some limitations. First, this was a retrospective study conducted at a single center. Second, selection bias could have influenced the enrolment of patients with LM occlusion, since most of them died before undergoing coronary angiography. Third, the control groups were derived from an angiographic database, which does not represent the total population with either LAD or LCX infarctions. In addition, a control group with acute coronary syndrome due to three-vessel coronary artery disease was not included, although the STE in lead aVR in this category resembled that of LM subtotal occlusion. Lastly, LM-AMI patient management changed over the study period, from thrombolytic therapy to using a stent, an intra-aortic balloon pump, and left ventricular assist devices; therefore, we did not evaluate prognostic differences resulting from different ECG patterns.

## Conclusion

This study described three main ECG patterns in LM occlusion according to the characteristics of STE: STEMI pattern, STE in aVR with diffuse ST depression, and STE in both aVR and aVL. These ECG patterns are demonstrated to be associated with different collateral filling territories. Compared with subtotal occlusion of the LM, LM occlusion presented with more STE in aVR and aVL, STEMI, LAFB, and LAFB + RBBB, prolongation of the QRS interval, and less STE in aVR and V1. The STE in the precordial leads from V2–V5, without V1, is a specific predictor for discriminating LM occlusion from LAD occlusion. The combination of two different STE criteria (STE in aVR and aVL and STE in I, aVL, and V2–V5 without V1) predicted LM occlusion with high specificity and moderate sensitivity, and the addition of fascicular block criteria (LAFB, LAFB + RBBB) further improved the specificity with some loss of sensitivity.

## Data availability statement

The original contributions presented in the study are included in the article, further inquiries can be directed to the corresponding author.

## Ethics statement

The studies involving human participants were reviewed and approved by Ethics Committee of Institute of Tianjin Chest Hospital. The patients/participants provided their written informed consent to participate in this study.

## Author contributions

The conception and design of the work and drafted the manuscript by CL and FY. The statistical work and interpretation of data were performed by JZ, YH, ZG, YL, and XL. This study was directed by HC. All authors contributed to the article and approved the submitted version.

## Conflict of interest

The authors declare that the research was conducted in the absence of any commercial or financial relationships that could be construed as a potential conflict of interest.

## Publisher's note

All claims expressed in this article are solely those of the authors and do not necessarily represent those of their affiliated organizations, or those of the publisher, the editors and the reviewers. Any product that may be evaluated in this article, or claim that may be made by its manufacturer, is not guaranteed or endorsed by the publisher.
